# Distribution of bioactive factors in human milk samples

**DOI:** 10.1186/s13006-019-0203-3

**Published:** 2019-02-11

**Authors:** Reka A. Vass, Agnes Kemeny, Timea Dergez, Tibor Ertl, Dora Reglodi, Adel Jungling, Andrea Tamas

**Affiliations:** 1 Department of Anatomy, MTA-PTE PACAP Research Group, Centre for Neuroscience, Medical School, University of Pécs, Pécs, Hungary; 2 Department of Pharmacology and Pharmacotherapy; Medical School, University of Pécs, Pécs, Hungary; 3 Department of Medical Biology and Central Electron Microscope Laboratory, Medical School, University of Pécs, Pécs, Hungary; 4 Institute of Bioanalysis, Medical School, University of Pécs, Pécs, Hungary; 5 Department of Obstetrics and Gynaecology, Medical School, University of Pécs, Pécs, Hungary

**Keywords:** Bioactive factors, Breast milk, Luminex, MDC, Flt-3L, Water phase, Lipid phase, Cytokines, Chemokines, Growth factor

## Abstract

**Background:**

Breast milk provides nutrition for infants and also contains a variety of bioactive factors that influence the development of the newborn. Human milk is a complex biological fluid that can be separated into different layers (water phase and lipid phase with its component water and lipid fractions). It can affect the developing human body along the whole length of the gastrointestinal tract, and through the circulation, its factors may reach every organ.

**Methods:**

In the present study, we analyzed milk samples collected monthly for 6 months from 16 mothers from the 4^th^ week postpartum between 2014 and 2016 in Baranya County, Hungary. The 96 samples provided us information about the fluctuation of certain bioactive factors during the first 6 months of lactation. We investigated with Luminex technology the concentrations of several cytokines (CD40, Flt-3L), chemokines (MCP-1, RANTES, GRO, MIP-1ß, MDC, eotaxin, fractalkine), and epidermal growth factor (EGF). Paired t-tests and one-way ANOVA followed by Bonferroni post-hoc tests were used to compare the data.

**Results:**

We detected the presence of each bioactive factor in every layer of the milk samples during the first 6 months of breastfeeding in widespread concentration ranges. In the case of GRO, MIP-1ß, MDC, Flt-3L, fractalkine, and eotaxin, the concentrations were constant during the first 6 months of lactation. The water phase of human milk contained higher factor concentrations compared to both fractions of the lipid phase for most factors (except eotaxin and MIP-1ß). The concentrations of CD40, EGF, MCP-1, and RANTES in the first 3 months were significantly different compared to the values detected between 4^th^ and 6^th^ months. In the water phase, the level of MCP-1 was significantly decreased, while all of the other factors increased during the 4^th^ through 6^th^ months. We found significantly higher EGF, GRO, and RANTES levels in the water fraction compared to the lipid fraction of the lipid phase.

**Conclusions:**

The novel findings of this investigation were the presence of Flt-3L and MDC in all layers of breast milk, and nearly all bioactive factors in the lipid phase. Due to their widespread physiological effects these factors may have an essential role in organogenesis.

## Background

Human milk is a complex biological fluid uniquely suited to the infant. Its quantity and composition are constantly changing during lactation and are influenced by numerous factors [1–3]. It promotes survival and development of the newborn due to its nutritional composition and non-nutritive bioactive factors, including growth factors, hormones and immunological factors, such as chemokines and cytokines with different physiological effects. The distribution and level of the bioactive factors vary widely in the milk [4, 5], and the exact role and effects of these factors during postnatal adaptation are still under investigation. These biologically active molecules are proven to protect the newborn from different infections affecting the respiratory and gastrointestinal tract [6, 7] and have an important role in gaining immunocompetence [8, 9]. In addition to the immunological effects they contribute to organogenesis and they are essential for the development and adequate functioning of the nervous, gastrointestinal and cardiovascular systems [10].

Bioactive components of human milk come from a variety of sources; some are produced and secreted by the mammary epithelium or by cells carried within the milk, while others are drawn from maternal serum and transferred across the mammary epithelium by receptor-mediated transport [10]. Previous studies have proven that human breast milk is segregable into water and lipid phases using different separation techniques. Preclinical and clinical studies have analyzed mainly the water phase of milk. However, breast milk has a complex lipid architecture [11] that forms a special biological colloidal system [12]. Typically, the fat content of mature human milk is about 4 g/dL. The lipid phase can be further divided into water and lipid fractions with additional separation techniques. The lipid fraction contains milk fat globules (MFG), secreted by the mammary epithelial cells; and their size varies from 0.2 to more than 15 μm [13]. The milk fat globules secreted into breast milk are made up of four layers (from inside, in order): a single layer of polar lipids and proteins, an entrained cytoplasm layer (10–20 nm wide) and a double layer of polar lipids, proteins, glycoprotein and cholesterol [14, 15]. The inner monolayer membrane originates from the endoplasmic reticulum, while the outer bilayer originates from the apical plasma membrane. The bioactive factors are found in the cytoplasm layer; this way they are protected during the transition through the gastrointestinal tract. Therefore, the contents of MFG are able to directly influence the composition of microbiota or colonization of the gut and also to absorb into the circulation [16, 17].

Only limited data are available about the content of bioactive factors during the first months of lactation [10]. Therefore, the aim of the present study was to examine the presence and changes in concentration of different bioactive factors during the first 6 months of lactation, which is the most critical period of postnatal adaptation and when exclusive breastfeeding is recommended. Our applied separation protocol allows us to separately analyze the composition of the water phase and the two fractions of the lipid phase involving the protected cytoplasm layer within the MFG. In our study we focused on the following bioactive agents in the three distinct layers: epidermal growth factor (EGF); cytokines: soluble cluster of differentiation 40 ligand (CD40), and FMS-like tyrosine kinase 3 ligand (Flt-3L); and chemokines: monocyte chemoattractant protein-1 (MCP-1), regulated on activation, normal T cell expressed and secreted (RANTES), macrophage inflammatory protein-1beta (MIP-1β), macrophage-derived chemokine (MDC), growth-regulated oncogene (GRO), eotaxin, and fractalkine. Cytokines and chemokines, such as CD40, MCP-1, RANTES, MIP-1β, and MDC play important roles in the modulation of immune responses and the regulation of immune cell migration [18–22]. Flt-3L is responsible for hematopoietic stem cell progenitor proliferation [23]. With this effect Flt-3L is involved in the development of the cardiovascular system similarly to GRO, which can stimulate endothelial proliferation during neovascularization [24]. Fractalkine and eotaxin are involved in the neurogenesis and the development of synaptic plasticity [25, 26]. Some bioactive factors can also help the adaptation of the gastrointestinal system, for example EGF enhances the differentiation and proliferation of enteral cells and the development of cell adhesions between hepatic and intestinal cells [27]. Since these factors have proven widespread effects, it would be beneficial to know their exact distribution in the three separated layers of human breast milk.

## Methods

### Study design

This was a descriptive study in which healthy mothers agreed to provide samples of breast milk for analysis. Data collection commenced at four weeks postpartum, and continued every four weeks until six months postpartum.

### Maternal inclusion criteria

Volunteers had delivered full-term infants by spontaneous active labor; pregnancy, labor, and postnatal adaptation were uneventful. Neither mothers nor infants had infectious diseases during the examined period. Mothers involved in the study took Elevit® multivitamin complex and folic acid tablets regularly; they had no special diet during pregnancy; all were nonsmokers. Participants – recruited with the help of health visitors of the Unified Health Institutions of Pécs – had normal prepregnancy and postpartum Body Mass Index (BMI) (Table 1). We included only mothers who were able to give milk samples at every time point of the investigation. The mothers used their own pumps to avoid transmission of infection.

**Table 1:** Characteristics of participants (*n* = 16)

Variables		
Maternal age (years), mean ± SD		27.8 ± 1.9
Range		25–32
Maternal weight (kg), mean ± SD		67.5 ± 3.4
Range		62–71
Parity		
	primipara	4
	multipara	12
Mode of birth		
	vaginal delivery	16
	Caesarean section	0

### Milk samples

Milk samples were collected between 2014 and 2016 at the Department of Obstetrics and Gynecology, University of Pécs and at the Unified Health Institutions of Pécs. We collected 5 ml of breast milk from mothers (*n* = 16) every fourth week during the first 6 months of lactation (*n* = 96 samples). The samples were hindmilk and were collected between 1 pm and 3 pm. Participants pumped the entire breast expression into a sterile bottle and 5 ml was poured into polypropylene tubes. We chose early afternoon as the target time for sample collection based on a previous report by Kent et al. [28] showing that milk fat content is lower in night and morning feedings compared to afternoon and evening feedings. Since we analyzed the bioactive content of the lipid layer, we speculated that samples obtained in the early afternoon might be close to representative of the average for all samples throughout a 24-h period. The collected fresh samples were stored at − 20 °C in sterile polypropylene tubes until analysis.

### Separation technique

In the present experiment, we applied different separation techniques to divide human milk into three different fractions from the original samples [11]. We segregated human milk into water and lipid phases, then the lipid phase was further subdivided into water and lipid fractions. To separate the samples into 3 different layers, milk was centrifuged at 4000 x g for 15 min at 13 °C to remove the pellet which contains the cells. This way we retrieved a water phase and a lipid phase, containing the milk fat globules. The lipid layer was suspended in 10 mM phosphate buffered saline (PBS) (pH 7.4) and stored at 4 °C overnight. Then it was churned with a Turrax at a medium speed until fat and sera were separated. After a brief warming at 38 °C, specimens were centrifuged at 100,000 x g for 1 h at 24 °C to obtain the water fraction and the lipid fraction. Then, the lipid fraction of the lipid phase was suspended in 10 mM PBS (pH 7.4).

### Luminex technology

Luminex Multiplex Immunoassay was performed to determine the protein concentrations of the following cytokines/chemokines using customized Milliplex Human Cytokine/Chemokine Magnetic Bead Panel (Merck Millipore, Burlington, USA): 1. EGF; 2. eotaxin; 3. Flt-3L; 4. fractalkine; 5. GRO; 6. MDC; 7. sCD40L; 8. MCP-1; 9. MIP-1ß; 10. RANTES. Standard curve range for each of these analytes was 3.2–10,000 pg/ml. Sensitivity (minimum detectable concentration in pg/ml) values were: EGF: 2.8; eotaxin: 4.0; Flt-3L: 5.4; fractalkine: 22.7; GRO: 9.9; MDC: 3.6; sCD40L: 5.1; MCP-1: 1.9; MIP-1ß: 3.0; RANTES: 1.2. Following previous optimizations, all samples were tested undiluted in a blind-fashion way and in duplicate. The experiment was performed according to the manufacturer’s instructions. Briefly, 25 μl of each sample (control and standard) was added to a 96-well plate (provided with the kit) containing 25 μl of capture antibody coated, fluorescent-coded beads. Biotinylated detection antibodies and streptavidin-PE were added to the plate after the appropriate incubation periods. After the last washing step, 150 μl of sheat fluid was added to the wells, the plate was incubated and read on the Luminex100 instrument. Five-PL regression curve were used to plot the standard curves for all analyte by the xPonent 3.1 software analysing the bead median fluorescence intensity. In the case of eotaxin, Flt-3L, fractalkine, MDC, and RANTES we eliminated from the analysis those data that were questionable because of the detection limits of the assays. In the case of RANTES, some of our data were above the sensitivity limit, but below the standard curve, so these results were calculated by extrapolation of the standard curve. Results are given in mean pg/ml ± standard deviation [SD] wet tissue.

### Statistical methods

When we compared the concentration values of the first 3 months to the results detected between 4^th^ and 6^th^ months we applied paired t-tests. One-way ANOVA followed by Bonferroni post-hoc tests were used to compare the concentration of the factors in the different layers during the first or second parts of the examination. Statistical analyses were performed by IBM SPSS Statistics v20.0 (IBM’s Corporate, New York, USA). Differences were considered to be significant when *p* values were < 0.05. All data are given as mean ± SD.

## Results

The mothers ranged in age from 25 to 32 years (Table 1). They ranged in weight from 62 to 71 kg; all had normal BMIs. Four mothers were primiparous, the others multiparous. All were delivered vaginally. During the first six months of lactation, we did not detect any time-dependent changes in the concentrations of GRO, MIP-1ß, MDC, Flt-3L, eotaxin, or fractalkine. The average values of these bioactive factors in the water phase and in the two layers of the separated lipid phase are shown in Table 2. We detected significantly higher levels of GRO, MDC, Flt-3L, and fractalkine in the water phase compared to both separated fractions of the lipid phase. No significant differences were detected between the concentration of MIP-1ß in the water phase and the separated fractions of the lipid phase. We measured significantly higher GRO concentration in the water fraction of the lipid phase compared to the lipid fraction. Eotaxin concentration in the water phase was similar to the lipid fraction, and it was not detectable in the water fraction. We did not detect significant differences between the separated fractions of the lipid phase in MIP-1ß, MDC, Flt-3L, or fractalkine (Table 2).

**Table 2:** Concentration of bioactive factors during the first 6 months of lactation [pg/ml]

Factors	Water phase (W)	Lipid phase (L)		p - values	
		Water fraction (L-WF)	Lipid fraction (L-LF)		
GRO	5398.36 ± 2250.44	1927.09 ± 563.33	652.36 ± 160.60	W vs L-WF W vs L-LF L-WF vs L-LF	< 0.0001 < 0.0001 0.0003
MIP-1ß	6.72 ± 2.58	5.35 ± 0.57	6.23 ± 1.44	W vs L-WF W vs L-LF L-WF vs L-LF	0.5280 1.0000 1.0000
MDC	21.48 ± 16.01	11.83 ± 8.15	12.25 ± 8.46	W vs L-WF W vs L-LF L-WF vs L-LF	0.0029 0.0069 1.000
Flt-3L	8.81 ± 2.36	6.25 ± 0	6.25 ± 0	W vs L-WF W vs L-LF L-WF vs L-LF	0.0235 0.0235 1.0000
eotaxin	9.62 ± 2.54	*Not detectable*	9.85 ± 3.89	W vs L-LF	0.9227
fractalkine	166.84 ± 81.77	43.29 ± 23.00	25.27 ± 7.13	WP vs L-WF WP vs L-LF L-WF vs L-LF	< 0.0001 < 0.0001 0.5774

*GRO* growth related oncogene; *MIP-1ß* macrophage inhibitory protein 1ß; *MDC* macrophage derived chemokine; *Flt-3L* Flt-3 Ligand

We found differences in CD40, EGF, MCP-1, and RANTES concentrations between the first 3 months and at 4–6 months of lactation. The concentrations of these factors in the water phase are presented in Table 3, and the factor content of the separated lipid phase is shown in Table 4. The CD40, EGF, and RANTES levels in the water phase increased significantly, and the MCP-1 level decreased significantly with time (Table 3). In the water fraction of the lipid phase, levels of all factors were significantly higher in the second (4–6 months) part of the examination period. In the lipid fraction of the lipid phase, MCP-1 and EGF increased with time, and CD40 and RANTES did not change significantly (Table 4).

**Table 3:** Concentration of bioactive factors presented in the water phase of human milk during the first 6 months of lactation [pg/ml]

Factors	Water phase [W]		*p*-values 1–3 vs 4–6 months
	1–3 months	4–6 months	
CD40	50.17 ± 2.76	65.77 ± 1.57	< 0.0001
EGF	2234.95 ± 790.45	4064.33 ± 953.71	< 0.0001
MCP-1	2986.49 ± 937.31	306.66 ± 146.57	< 0.0001
RANTES	3.0 ± 0.75	5.18 ± 1.56	0.0273

*CD40* cluster of differentiation 40; *EGF* epidermal growth factor; *MCP-1* macrophage chemokine protein-1; *RANTES* regulated on activation, normal T cell expressed and secreted

**Table 4:** Concentration of bioactive factors presented in the lipid phase of human milk during the first 6 months of lactation [pg/ml]

Factors	Lipid phase [L]					
	Water fraction [L-WF]			Lipid fraction [L-LF]		
	1–3 months	4–6 months	*p*- values 1–3 vs 4–6 mo	1–3 months	4–6 months	*p*- values 1–3 vs 4–6 mo
CD40	22.87 ± 7.36	34.11 ± 6.67	0.019	22.98 ± 7.87	29.80 ± 0.01	0.1330
EGF	828.48 ± 480.11	1851.26 ± 456.69	< 0.0001	375.25 ± 129.18	669.11 ± 197.70	< 0.0001
MCP-1	13.02 ± 3.49	21.41 ± 9.71	0.0245	5.07 ± 0.69	10.67 ± 6.01	0.0020
RANTES	3.13 ± 0.73	4.34 ± 0.59	< 0.0001	3.65 ± 1.86	2.37 ± 0.0	0.2031

*CD40* cluster of differentiation 40; *EGF* epidermal growth factor; *MCP-1* macrophage chemokine protein-1; *RANTES* regulated on activation, normal T cell expressed and secreted; *mo* months

**Fig. 1:**
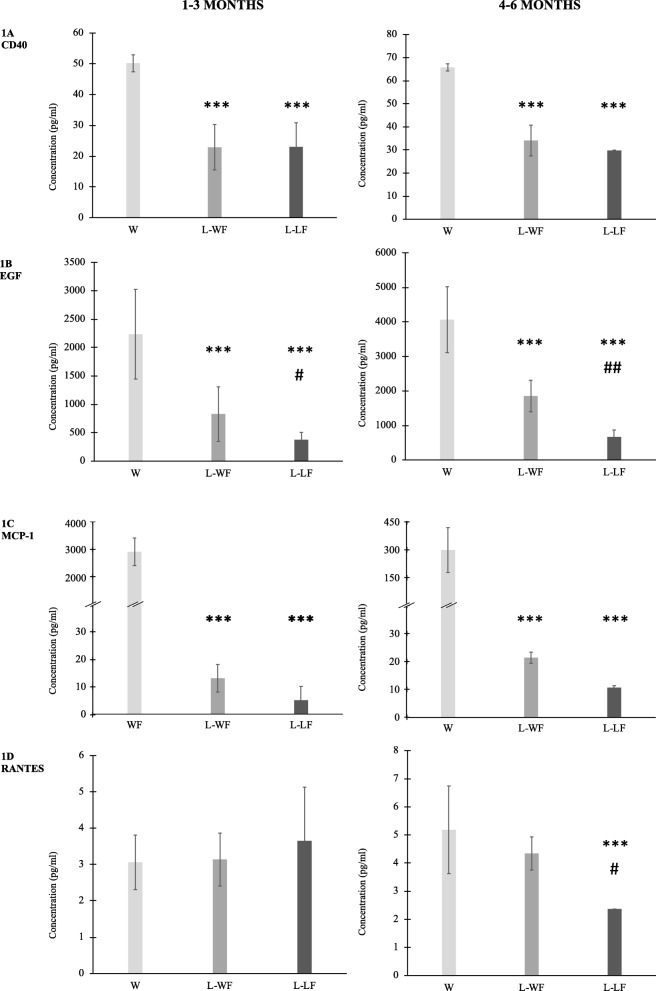
Concentration of CD40, RANTES, MCP-1 and EGF between the 1–3 and 4–6 months of lactation in the three separated layers of breast milk: water phase [W], water fraction of lipid phase [L-WF] and lipid fraction of lipid phase [L-LF]. 1A: CD40; 1B: EGF; 1C: MCP-1; 1D: RANTES. **a** CD40 ***P < 0.0001 vs W, **b** EGF ***P < 0.0001 vs W, ##*P* < 0.01 vs L-WF, #*P* < 0.05 vs L-WF, **c** MCP-1 ***P < 0.0001 vs W, **d** RANTES ***P < 0.0001 vs W, #P < 0.05 vs L-WF. Note. CD40 = cluster of differentiation 40; MCP-1 = macrophage chemokine protein-1; RANTES = regulated on activation, normal T cell expressed and secreted; EGF = epidermal growth factor

In the case of four factors – CD40, EGF, MCP-1, and RANTES, − we detected significant changes between the different layers of breast milk during the first (1–3 months) and the second (4–6 months) parts of the examination period. Comparing to the three layers of breast milk, the water phase contained significantly higher levels of CD40, EGF, and MCP-1 during the first 3 months. We detected significantly higher EGF concentration in the water fraction of the lipid phase compared to the lipid fraction of lipid phase in the first 3 months of lactation. In the case of CD40, MCP-1, and RANTES we did not measure significant differences between the two layers of the lipid phase during the first 3 months of breastfeeding. During the second part of the examination period, we detected significantly higher CD40, EGF, and MCP-1 concentrations in the water phase compared to the water fraction of the lipid phase. In all cases, the water phase contained significantly increased levels of factors compared to the lipid fraction of lipid phase. When we compared the two layers of the lipid phase, the water fraction of the lipid phase contained significantly higher EGF and RANTES concentrations compared to the lipid fraction of the lipid phase. We did not find differences in CD40 or MCP-1 level between the two layers of lipid phase between the 4^th^ and 6^th^ months (Fig. 1).

## Discussion

In the present study, we detected several bioactive factors during the first 6 months of lactation in the three separated layers of human milk: in the water phase and in the water and lipid fractions of the lipid phase. Presumably each of these layers has its own role during organogenesis. The content of the water phase affects the infant’s body through mucosal absorption from the oral cavity. The bioactive components in the MFG, because of the protective lipid layer, directly affect the lower gastrointestinal tract, and through the circulation they may affect the developing human body [10]. Cytokines and chemokines are small soluble factors that modulate biological signals with remarkable capabilities, such as influencing growth and development, hematopoiesis, and the immune system through lymphocyte recruitment, T cell subset differentiation and inflammation [29, 30].

During postnatal adaptation, the gastrointestinal tract becomes more actively involved in enteral nutrition [31]. Several research groups have proven that the microbiome in infants is an important regulator of developing subsistence. The human microbiome is a constantly changing system influenced by hormones, cytokines, and chemokines. Its development starts at the moment of birth, and the most important changes occur during the first year of life [32–35]. The connections between the brain and gut have been investigated previously; this communication axis bound by the vagus nerve is described as the microbiota-gut-vagus-brain axis [36]. Studies have proven the connection of the microbiome to disorders such as visceral pain, autism spectrum disorder, cardiovascular risk, obesity, depression, and sclerosis multiplex [36]. Previous studies suggest that the gut microbiome modulates molecular mechanisms that influence allergic reactions [37].

The composition of human milk influences the developing immune system in the early, critical period of postnatal adaptation [38]. Cytokines and chemokines in breast milk reach the neonatal intestines intact due to the protection from digestion by protease inhibitors, such as alpha 1-antichymotrypsin and alpha 1-antitrypsin, which are present in human milk. In addition, the gastric digestion of proteins is reduced during the first 3 months of life because of the limited secretion of pepsin and hydrogen ions and the general immaturity of the newborn’s digestive abilities. Therefore, these factors can exert their biological activities in their intact form [39, 40].

Several cytokines are known to play important roles in the brain, because they are transported across the blood-brain barrier [41]; the rate of transport varies among regions and is affected by cellular components of the blood [42]. The cytokines and chemokines affect cellular and molecular processes of hippocampal-dependent long-term memory consolidation and neurogenesis [43]. Chemokines are involved in neurodevelopment and neurophysiological signaling, and they attract leukocytes and mediate immune responses in the brain [44].

GRO is a small cytokine belonging to the CXC chemokine family that was previously called GRO1 oncogene, and now it is called CXCL1. GRO is expressed physiologically during protective reactions against microbiological agents or tumor progression [45]. GRO’s chemoattractant function in neutrophil granulocytes has been described earlier [18] and it is also known as a mediator of inflammatory responses [46]. We detected high amount of GRO in every phase of breast milk as Radillo and coworkers found in whole milk samples [47]. It is suggested that GRO may moderate the development of gut-associated lymphoid tissue. In neovascularization GRO is necessary for ideal endothelial cell proliferation [24]. Hence, it may directly affect the vascularization of the intestinal wall and through absorption also the extraintestinal vessels of the body.

Eotaxin, a member of the CC chemokine subfamily, was present in low concentration in the water phase of human milk. Previous examinations proved its role in neurogenesis [25] and plasticity [48]. Other investigators reported higher concentration of eotaxin in colostrum and in the first week of lactation [49, 50]. It is possible that the concentration of eotaxin fluctuates with time since in our examination this factor was present in low concentration in every layer. Although it is able to be transported through the blood-brain-barrier [41], this factor in these low concentrations may have little effect on the development of the brain.

A constitutively expressed chemokine named fractalkine in humans and neurotactin in mice [51] is a member of the CX_3_C chemokine family. Experiments pointed out that fractalkine promotes synaptic plasticity in the developing nervous system. It modulates synaptic transmission and has long-term potentiation in the hippocampus [26]. Fractalkine is upregulated in the hippocampus; it also regulates spatial orientation learning processes. Based on our work, fractalkine is present in higher concentrations in the different milk layers than what others have reported in whole milk [52]. In summary, fractalkine reaches different parts of the developing human body through absorption and it may modulate neurodevelopment.

The monocyte chemoattractant protein 1 (MCP-1) or CCL2 **(**chemokine [C-C motif] ligand 2) is an inducible cytokine. It is produced by endothelial and epithelial cells, and fibroblasts and induced by oxidative stress, growth factors, and other cytokines [53, 54]. MCP-1 regulates the migration of monocytes, natural killer (NK) cells, and memory T-lymphocytes [21]. During the first 3 months of lactation, we detected high levels of MCP-1, which dramatically decreased from the 4^th^ month; we suggest that this factor particularly plays a role in the early adaptation of immune system as an influencer of T cell immunity [55–57]. This protein was reported to be present in different concentration in colostrum and mature milk samples [49, 50, 58–60]. Our results show that the water phase contained an order of magnitude higher level of MCP-1 compared to the lipid phase, especially in the first 3 months of lactation. We hypothesize that the presence of this factor influences the early postnatal adaptation of the immune system, mainly the gut-associated lymphatic tissue (GALT).

One of the selected chemokines, RANTES, modulates the homing and trafficking of monocytes and T-cells. The presence of RANTES in non-lactating mammary epithelial cells was proven with immunohistochemistry [60]. It plays a role in chemotaxis and also promotes leukocyte infiltration during inflammation [61]. Previous examinations measured elevated values in early lactation [49, 58, 62]. Since in breast milk we detected RANTES only in low concentrations, we suggest that it has little immunological impact.

Two of the examined factors were cytokines, CD40 and Flt-3L. CD40 is expressed by a variety of cells, such as monocytes, B-cells, antigen presenting cells, endothelial cells, smooth muscle cells, and fibroblasts [63]. Presumably, the maternal T-cells interact with B cells of the newborn and stimulate the immune system because T-cells of infants are immature. Presumably, CD40 plays a role in the immunological adaptation of the adequate immune responses to unknown agents.

We also examined the growth factor EGF**,** which was present in one of the highest concentrations among the analyzed bioactive factors in the 3 layers of human milk. The level of EGF in colostrum is 2000 times higher than in maternal serum and 100 times higher than in foremilk [27]. Based on previous examinations EGF is resistant against low pH and digestive enzymes. Therefore, the water phase of milk may reach the surface of epithelium in the whole length of the gastrointestinal tract. Due to immature cell connections of the epithelium, EGF can spread into the circulation [64, 65]. EGF stimulates DNA synthesis of enterocytes and enables their differentiation [66, 67].

To our knowledge, no data exist about the presence of Flt-3L and MDC in breast milk. Lyman and coworkers previously proved that Flt-3L stimulates the primitive hematopoietic stem cell progenitors’ proliferation [23] and it also promotes T cell activation [68]. Since this factor can be absorbed into the circulation, the question is raised whether it may participate in the development of the hematopoietic system and in the development of adaptive immunity.

One of the constitutively expressed chemokines is MDC, the expression of which is enhanced by lipopolysaccharides and primary proinflammatory cytokines in macrophages [69]; it is also produced by thymus epithelial cells and expressed by the hematopoietic system [70]. The production of MDC has been observed in Th1 and Th2 cells [20], and it plays a role in type II reactions in atopic dermatitis in humans [71]. We measured the chemokine MIP-1ß in low concentration as in a previous study [72]. MIP-1ß is crucial for the development of appropriate immune responses and the mediation of inflammation [22]. It is a potent chemoattractant of T lymphocytes and attracts exclusively activated T-cells [73]. In human milk, MDC and MIP-1ß are presumably playing important roles in the adaptation of immune system through the differentiation of T cells and may influence the development of T-cell mediated immune responses.

## Conclusions

Milk provides not only the building blocks for development but also the endocrinological and hormonal signals that contribute to the biophysiological organization of the infant. The separation of human milk into different fractions enables analyzing the presence and concentration of various bioactive factors that may be present in low concentrations in human milk yet have significant biological effects. The present study showed that the water phase is not the only influential part of breast milk, but the MFGs in the lipid layer contain important bioactive compounds. Our analysis provides a more complete picture of the fluctuating levels of factors in breast milk since we measured the composition of breast milk through the first 6 months of life. Based on our work, EGF and the analyzed cytokines and chemokines are present in breast milk during the first 6 months of lactation, including Flt-3L and MDC, which have not previously been reported as present in breast milk. These agents may be absorbed from the oral cavity from the water phase of human milk and they can spread to every organ of the infant. Factors present in the lipid fraction are protected, so they are able to reach the lower gastrointestinal tract where they may have a local effect on gut mucosa or systemic effects through absorption into the circulation. Further research will be required to detect the concentration ranges within which these factors are essential and beneficial for the development of newborns.

## Abbreviations

BMI: Body Mass Index
CCL2: Chemokine (C-C motif) ligand 2
CD40: Soluble cluster of differentiation 40 ligand
CXC, CXCL1, CC, CX_3_C: Chemokine subfamilies
EGF: Epidermal growth factor
Flt-3L: FMS-like tyrosine kinase 3 ligand
GALT: Gut-associated lymphatic tissue
GI: Gastrointestinal tract
GRO: Growth-regulated oncogene
MCP-1: Monocyte chemoattractant protein-1
MDC: Macrophage-derived chemokine
MFG: Milk fat globules
MIP-1β: Macrophage inflammatory protein-1beta
PBS: Phosphate buffered saline
RANTES: Regulated on activation, normal T cell expressed and secreted
